# Aquagenic palmar keratoderma associated with palmoplantar hyperhidrosis: a case report

**DOI:** 10.1093/omcr/omag001

**Published:** 2026-02-18

**Authors:** Marah Mansour, Dyala Sayed Ahmad, Zakaria Ismail, Dana Almamsani, Lutfallah Raffoul, Eilaaf Altamer, Wissam Georgeos

**Affiliations:** Faculty of Medicine, Tartous University, 8th March st, Mustafa mosque, Tartous, Syria 0000, Syrian Arab Republic; Division of Colon and Rectal Surgery, Department of Surgery, Mayo clinic, Rochester, MN, United States of America; Department of Dermatology, Tishreen University Hospital, Latakia center, Latakia, Syria, Syrian Arab Republic; Faculty of Medicine, Damascus University, Al-Rawda District, Jaramana, Rif Dimashq Governorate, Syria, Syrian Arab Republic; Faculty of Medicine, Damascus University, Al-Rawda District, Jaramana, Rif Dimashq Governorate, Syria, Syrian Arab Republic; Faculty of Medicine, Al Andalus University for Medical Sciences, Al Andalus Street — Al Qusoor District — Tartous — Syria, Syrian Arab Republic; Department of Internal Medicine, Damascus hospital, Damascus center, Damascus, Syria, Syrian Arab Republic; Faculty of Medicine, Al Baath University, Homs _ Al-Nezha _ Al-Jazeera Street, Syrian Arab Republic

**Keywords:** Aquagenic palmoplantar keratoderma, cystic fibrosis, palmoplantar hyperhidrosis, palmar thickening, treatment, case report

## Abstract

Aquagenic palmoplantar keratoderma is a rare dermatological condition characterized by mild palmar thickening and burning pain upon exposure to water. A 31-year-old female with aquagenic keratoderma of the palms associated with palmoplantar hyperhidrosis presenting with wrinkling and hyperkeratosis within minutes of hand contact with water, resolving quickly after drying. While cystic fibrosis is a known association, most cases remain idiopathic. Treatment options such as 20% aluminum hydroxide, aluminum chloride (15%–20%), urea, salicylic acid, and botulinum toxin can be challenging, with the patient being evaluated after treatment with 16% aluminum chloride.

## Introduction

Aquagenic palmoplantar keratoderma (APPK) is a rare dermatological condition first described in 1996 [1, 2]. Onset is typically during the second decade of life, with a preference for females. Observed hereditary or sporadically in 50% of cystic fibrosis (CF), 10%–25% of Cystic Fibrosis Transmembrane Conductance Regulator (CFTR) mutation heterozygous carriers [1, 3], and patients with marasmus, Raynaud disease, hyperhidrosis, and atopic dermatitis [4]. It is also linked to cyclooxygenase-2 (COX-2) inhibitors and aspirin use [5]. Notably, COX-2 inhibitors reduce prostaglandin synthesis, leading to increased sodium retention in epidermal cells, similar to their effect on kidney cells. Aspirin, as a nonselective COX inhibitor, may have a different mechanism of action, potentially contributing to barrier dysfunction and altered skin hydration. Clinically characterized by thickening and white to translucent, ‘pebbly’ changes on the palms shortly after immersion in water within three minutes (early onset of symptoms), associated edema and burning pain disappear shortly after drying the hands. Diagnosis is made on patient history and physical exam [6, 7]. In dermoscopy, the papular lesions are at sites of dilated acrosyringeal ostia. Histologically, normal skin or dilated eccrine ostial and a mildly hyperkeratotic stratum corneum may be seen. Differential diagnoses are aquagenic pruritus, aquagenic urticaria, and hereditary papulotranslucent acrokeratoderma. Treatment options involve topical 20% aluminum hydroxide, urea, salicylic acid, and botulinum toxin. Complications are hyperhidrosis and CF.

## Case presentation

**Figure 1:**
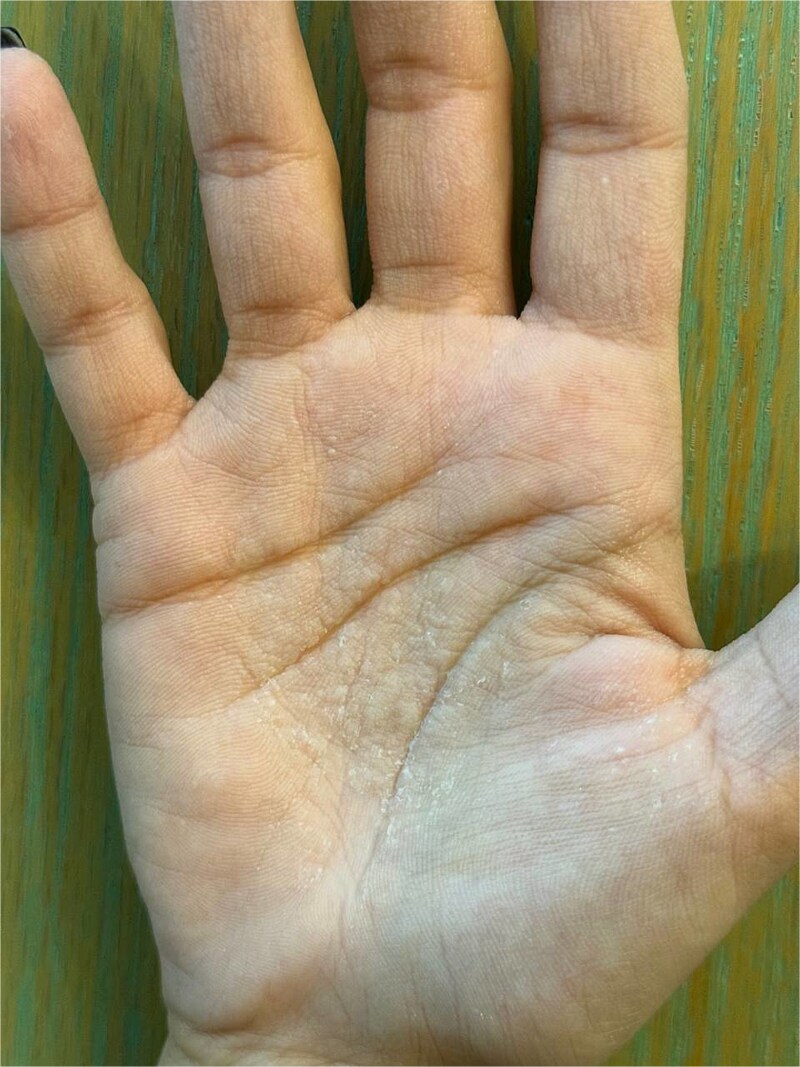
Hyperkeratosis and edema with translucent whitish cobblestone papules on palms, more obvious on the right one, after 3 minutes of immersion in water (positive hand in the bucket sign).

A 31-year-old female patient presented to the Dermatology Department with exaggerated wrinkling and hyperkeratosis, with itching and pain developing on palms after brief contact with water and fading away after drying hands. The symptoms started to happen for three years within less than ten minutes after exposure to water. This period decreased to just 2–3 minutes after exposure, and the pain sensation has exacerbated, especially on the right palm. However, soles are not involved. Medical history includes palmoplantar hyperhidrosis since childhood, migraine for one year, and surgery for endometriosis management six years ago. Medication history includes oral contraceptive pills (combined norethindrone and estradiol) for 6 years, and ibuprofen for migraine attacks. No personal or familiar history of CF was reported. No smoking or alcohol consumption was recorded. On physical examination, the palms were sweaty with mild hyperkeratosis, and translucent whitish cobblestone papules. Following immersion in water for three minutes, papules become more prominent in addition to wrinkling associated with edema, and burning sensation (Fig. 1), consistent with a positive (hand in the bucket sign). The findings disappeared spontaneously after drying hands. This clinical response suggested the diagnosis of APPK. After using topical aluminum chloride 16% one time daily, the symptoms improved in a few weeks but then relapsed again.

## Discussion

APPK, which is also known as transient reactive and acquired papulotranslucent acrokeratoderma [7] is a rare condition [2, 4–7], and was first described in 1996 [3]. It is most common in young adult women and female adolescents [2, 3]. The etiology is still unknown [2]. Many reports are related to heredity as an autosomal recessive hereditary dermatosis [3], but in our case, no familial conditions were observed. Some studies suggested the connection of abnormal sweat gland function [5], and it tends to be associated with 44%–80% of CF patients and 25% of CF carriers, which reveals the strong relationship between APPK and CF. Based on this, some studies recommended a seven-minute hand test that involves a brief immersion in water as an efficient screening method for CF patients [4]. Notably, our patient did not have CF, but the possibility of being a carrier has not been excluded. In addition, some studies described patients with drug-induced APPK (Aspirin, celecoxib, and rofecoxib). These medications increase sodium retention of epidermal cells because of their COX-2 inhibition ability, which may lead to + sodium reabsorption similar to the effect on kidney cells [5]. Aquaporins (AQPs) are integral membrane proteins that regulate water transport in various tissues, including the kidneys and epidermis. In the kidneys, COX-2 inhibition is known to alter prostaglandin synthesis, which in turn affects sodium and water homeostasis by modulating AQP expression and function. A similar mechanism may occur in epidermal cells, where altered AQP expression could contribute to abnormal water retention and increased skin fragility in APPK. This dysregulation may partially explain the pathophysiological changes observed in patients with COX-2 inhibitor-associated APPK [10]. Meanwhile, our patient had oral contraceptive pills (OCPs) (combined Norethindrone and Estradiol), which is not a COX-2 inhibitor, since 2017, and ibuprofen for migraine attacks, which started 1 year ago. Some cases involved the nose, mouth, upper lip, double ankle joints, and calves in addition to the common places like the palms and soles. However, our case involved only the palms and had palmoplantar hyperhidrosis history. Palmoplantar hyperhidrosis, nephrotic syndrome, marasmus, and cardiac anomalies may be associated with APPK [1]. Clinically, patients usually present with exaggerated wrinkling of the palms, and more rarely the soles, after a brief immersion in water for 10 minutes to a few hours. And after minutes to hours of drying the hands, this wrinkling often disappears. In addition to pain, burning, itching, and tingling could be presented [1]. Notably, the symptoms appeared only 2–3 minutes after the contact with water and were more significant on the right palm in our patient. Physical examination usually reveals translucent to whitish papules on the palms after exposure to water, which is also known as the ‘hand-in-the-bucket’ sign [8]. In our case, the patient had similar findings with mild hyperkeratosis and sweaty palms, especially the right one. Classically, the diagnosis depends on the clinical history and physical exams. A ‘hand-in-the-bucket’ test is usually performed to reproduce the palmar eruption, and that involves immersing the hands in 15°C water for five minutes. A biopsy can confirm the diagnosis, demonstrating dilated acrosyringeal ostia, mild hyperkeratosis, spongiosis, focal acanthosis, and perivascular lymphocytic infiltration in some cases [1]; however; the diagnosis was established depending on the clinical and physical findings in our case. Patients typically seek assistance because they are physically or socially distressed, even though APPK is benign and typically asymptomatic [9]. So far, there is no effective treatment for APPK. The most common treatment is topical 20% aluminum hydroxide, either alone or combined with a keratolytic preparation that consists of salicylic acid or topical urea. In addition, many botulinum toxin treatments were managed with good outcomes. Alternative medications, which include antihistamines and topical steroids, may be provided with small benefits [1]. However, our patient was administered external use of 16% aluminum chloride one-time daily with mild symptomatic improvements which is applied topically to the affected areas, where it functions as an astringent by obstructing sweat gland ducts and reducing moisture-related hyperkeratosis. This mechanism is particularly relevant in APPK, as it minimizes water retention in the stratum corneum. In conclusion, APPK is a rare idiopathic dermatological condition that may be isolated or associated with other diseases, most notably CF. Diagnosis is often made clinically. Currently, many symptomatic treatments can be applied. However, we still need more future research on the exact pathogenesis of this condition so that we can manage it effectively.

## Supplementary Material

Derma-Letter_response(2)_omag001

## Data Availability

Not applicable. All data (of the patient) generated during this study are included in this published article and its supplementary information files.
