# Supplementary material for: Aquagenic palmar keratoderma associated with palmoplantar hyperhidrosis: a case report
Source: Oxf Med Case Reports. 2026 Feb 18;2026(2):omag001. doi: 10.1093/omcr/omag001 (PMC12916001; doi:10.1093/omcr/omag001)
Supplement: Derma-Letter_response(2)_omag001 [file derma-letter_response(2)_omag001.docx]

**Response letter :**

1.Introduction – Terminology Adjustment

Agreed and edited as required in the introduction section page number 2 . We have modified the phrase to “females” to maintain conciseness and inclusivity in referring to the affected population.

2. Introduction – Expand on COX-2/Aspirin Link

Response: We have agreed and expanded the discussion on COX-2 inhibitors and aspirin page number 2, emphasizing that COX-2 inhibitors reduce prostaglandin synthesis, leading to increased sodium retention in epidermal cells, similar to their effect on kidney cells. Aspirin, as a nonselective COX inhibitor, may have a different mechanism of action, potentially contributing to barrier dysfunction and altered skin hydration.

3. Discussion – Separate COX-2 and Aspirin Analysis.

Response: We have separated the discussion of COX-2 inhibitors and aspirin, clarifying their distinct mechanisms. While COX-2 inhibitors (e.g., celecoxib) selectively inhibit COX-2, reducing prostaglandin-mediated sodium retention, aspirin inhibits both COX-1 and COX-2, potentially affecting epidermal barrier function through multiple pathways.

4. Discussion – Dysregulation of Aquaporins in Sodium Reabsorption

Response: Agreed and expanded the discussion on the dysregulation of Aquaporins page number 3.

Aquaporins (AQPs) are integral membrane proteins that regulate water transport in various tissues, including the kidneys and epidermis. In the kidneys, COX-2 inhibition is known to alter prostaglandin synthesis, which in turn affects sodium and water homeostasis by modulating AQP expression and function. A similar mechanism may occur in epidermal cells, where altered AQP expression could contribute to abnormal water retention and increased skin fragility in APPK. This dysregulation may partially explain the pathophysiological changes observed in patients with COX-2 inhibitor-associated APPK

5. Discussion – Remove Unnecessary COX-2 Comment Regarding OCPs

Response: Agreed and edited as required in the discussion section page number 3, as it does not add relevant information to the discussion.

6. Discussion – Expand on Biopsy Findings

Response: We have expanded this section to include histopathological features, such as: dilated acrosyringeal ostia, mild hyperkeratosis, spongiosis, focal acanthosis, and perivascular lymphocytic infiltration in some cases.

7. Was a DLQI score done? Can these patients go on with activities of daily living such as washing dishes, showering, etc.?

The Dermatology Life Quality Index (DLQI) score was not assessed in this case. However, we acknowledge its importance in evaluating the impact of aquagenic palmar plantar keratoderma (APPK) on quality of life. While many patients can perform daily activities such as washing dishes and showering, these tasks may exacerbate symptoms, causing discomfort and functional impairment. similar to our patient case.

8. If space allows, it is worth expanding briefly on how aluminum chloride 16% is administered and its mechanism of action.

We appreciate this suggestion. Aluminum chloride 16% is applied topically to the affected areas, where it functions as an astringent by obstructing sweat gland ducts and reducing moisture-related hyperkeratosis. This mechanism is particularly relevant in APPK, as it minimizes water retention in the stratum corneum. We will include this information in the manuscript.

9. It may be worth clarifying the diagnostic criteria when it comes to timing (as in the introduction you define “within three minutes,” but in the discussion, the hand-in-the-bucket test is done for “five minutes”).

The “within three minutes” timeframe in the introduction refers to the early onset of symptoms, while the “five minutes” in the discussion pertains to the standardized duration of the diagnostic immersion test. We will revise the wording for clarity.

Response to Reviewer 2

1. The authors described that the patient had baseline skin changes on the hands even before water exposure. Are there before-and-after photos to demonstrate the changes?

Unfortunately, before-and-after photographs were not obtained for the same hand, but we have a comparison photographs of both hands. One hand after the immersion and the other without.

2. Did the patient undergo genetic testing? How was cystic fibrosis (CF) or a carrier state excluded?

Genetic testing was not performed in this case. While the clinical presentation was not suggestive of cystic fibrosis, we acknowledge that genetic testing would have been useful to definitively exclude CF or carrier status.

3. The authors mentioned that COX-2 inhibition can be associated with APPK and that the patient had been taking ibuprofen. Was the patient advised to stop taking ibuprofen, and if so, what was the response?

The patient was not advised to discontinue ibuprofen during the evaluation. Given the potential link between COX-2 inhibition and APPK, discontinuing ibuprofen could have provided valuable insight into its role in disease progression.

4. The discussion is quite disorganized. For example, Pg 3, lines 28-29 have no connection to the sentences before or after. There are also no breaks or appropriate use of paragraphs. I suggest that the discussion be re-written in a more organized and readable fashion with the use of appropriate paragraphs. There is also repetitive information about the “hand-in-the-bucket” sign, which can be consolidated with better organization.
